# RETRACTION: Exploring the Comorbidity Mechanisms Between Psoriasis and Obesity Based on Bioinformatics

**DOI:** 10.1111/srt.70290

**Published:** 2025-11-19

**RETRACTION**: C. Gao, and J. Li, “Exploring the Comorbidity Mechanisms Between Psoriasis and Obesity Based on Bioinformatics,” *Skin Research and Technology *30, no. 2 (2024): e13575, https://doi.org/10.1111/srt.13575.

The above article, published online on 26 January 2023 in Wiley Online Library (wileyonlinelibrary.com), has been retracted by agreement between *Skin Research and Technology*; and John Wiley & Sons Ltd. The article was accepted for publication following an insufficient peer review process that does not meet the standards of the journal's peer review policy. Furthermore, several flaws and inconsistencies between results presented and conclusions drawn were identified. Finally, the article presents numerous citations that cannot be traced resulting in it lacking adequate support from the literature. Accordingly, the article has been retracted. The authors have been informed of the retraction decision but were unavailable for final confirmation.

